# Navigating life’s twists and turns: characteristics of life events across adulthood

**DOI:** 10.1007/s10433-025-00838-0

**Published:** 2025-01-20

**Authors:** Sonja Radjenovic, Fiona S. Rupprecht, Jana Nikitin

**Affiliations:** Department of Developmental and Educational Psychology, University of Vienna, Liebiggasse 5, 1010 Vienna, Austria

**Keywords:** Anticipation, Age normativity, Perceived control, Valence, Life circumstances, Age differences

## Abstract

This study examines whether there are age-related differences in the experience of life events across adulthood. We hypothesized that older adults would report life events that are less anticipated, less normative, less controllable, less positive, and more strenuous than younger adults due to increasing developmental losses and decreasing gains. We investigated how age (linear, quadratic, and cubic) relates to life-event characteristics by comparing different events and analyzing the same events across individuals, to distinguish between the effects observed across life events and those that emerge after accounting for the specific life event in question. Additionally, we hypothesized that older adults would cope better with less favorable events due to their life experience and emotion regulation skills. Analyses of 6,688 participants (18–90 years) showed that while older adults reported less favorable life events, they coped better with the same events than younger adults. The results underscore the importance of distinguishing between age and life event effects. They also show that life-event characteristics are consistently linked to well-being throughout adulthood.

**Supplementary Information:**

The online version contains supplementary material available at 10.1007/s10433-025-00838-0.

Significant life events – events that are “clearly timed, disrupt one’s everyday routine, and are perceived as personally significant and memorable by those who experienced them” (Luhmann et al. 2021; p. 2) – have per definition a significant impact on people’s lives. However, little is known about how the characteristics of life events change over the course of adult life and how these characteristics affect individuals’ well-being, subjective health, and life satisfaction in an age-dependent manner. To answer these questions, the present study investigates whether the characteristics of significant life events differ with age, that is, whether life events are experienced differently in terms of (a) anticipation, (b) age normativity, (c) perceived control, (d) valence, and (e) strain across the adult lifespan (Luhmann et al. 2021). In addition, we examine whether these characteristics differentially predict subjective well-being, subjective health, and life satisfaction in younger and older adults. We define subjective well-being as the affective component of well-being throughout the manuscript. The results of the study will help understand how people’s lives are shaped by significant life events, and how individuals respond and adapt to life events across adulthood.

## Age differences in characteristics of significant life events

Characteristics of significant life events are typically derived from empirical assessments. Luhmann et al. (2021) discuss two main approaches: the typological approach (e.g., categorizing events as “positive” or “negative”) and the dimensional approach (where characteristics vary on a continuous scale). The dimensional approach is more commonly used in literature. Prominent examples of dimensional approaches include 1) DIAMONDS (an empirically-derived taxonomy of major dimensions of situation characteristics; Rauthmann et al. 2014), 2) CAPTION (a lexically-derived taxonomy of psychological situation characteristics; Parrigon et al. 2017), and 3) life-event characteristics by Luhmann and colleagues (2021). Our approach aligns with Luhmann et al. (2021), while also drawing on other relevant studies to identify key aspects of life events.

We focus on three core dimensions: (1) *temporal aspects* (i.e., timing of an event), including the anticipation and age normativity of life events (Luhmann et al. 2012); (2) *perceived control*, reflecting the agentic capacity to influence events (Lang and Heckhausen 2001) and (3) *emotional significance*, with valence and strain representing distinct facets of emotional response. Valence captures the overall positive or negative emotional experience of an event, while strain reflects the challenge or stress it poses (Luhmann et al. 2021). In the following, we describe hypotheses for each of these characteristics.

*Anticipation* refers to the extent to which a person can foresee or predict the occurrence of a particular life event before it happens (Luhmann et al. 2012). Life events vary in terms of anticipation. Some events, such as the birth of a child or marriage, come with a high level of anticipation. Other life events may be unexpected, such as the sudden death of a loved one. As time passes, life events that are less anticipated may become more prominent (Wrosch and Freund 2001). This might be because losses, which are more common among older adults (Kaspar et al. 2023), are generally less anticipated. With age, the range of possible life events with uncertain timing expands, which may make it more challenging to anticipate specific upcoming events. For example, health decline can coincide with various other changes, such as the loss of social connections, the passing of loved ones, or adjustments in living circumstances (e.g., moving to a care facility). This unpredictability may make it harder for older adults to foresee which life events may happen next.

*Age normativity* refers to the socially shared expectations about the sequence of life events that individuals typically experience from birth to death. Normative life events are likely at a specific age (e.g., childbirth, retirement); non-normative events are less frequent and can occur at any age (e.g., the end of a relationship, relocation; Wrzus et al. 2013). Normativity varies with age. For instance, starting a family is more typical in young adulthood, while the loss of a partner is more associated with older adulthood (Wrosch and Freund 2001). Whereas adolescence, young, and middle adulthood are characterized by relatively strong normative expectations regarding the timing of life events (e.g., starting a new romantic relationship, achieving job promotions), older age is less structured by such norms (Freund et al. 2009). In older adulthood, non-normative events might accumulate and become more prominent (Wrosch and Freund 2001). Additionally, older adults have typically experienced a greater number of life events throughout their lives.

*Perceived control* refers to an individual’s subjective sense of having the power to shape one’s actions, decisions, and the resulting consequences (Lang and Heckhausen 2001). Research findings indicate a pattern of increasing perceived control during early adulthood, followed by periods of stability and decline (Mirowsky and Ross 2007). Perceived control tends to decrease in situations involving losses (Lachman 2006). Many of these losses accompany aging, such as biological changes, health problems, and bereavement (Lachman et al. 2011). As a result, older adults might perceive life events as less controllable than younger adults.

The *valence* of life events refers to the experience of the life event as either positive (e.g., the birth of a child) or negative (e.g., the loss of a loved one; Luhmann et al. 2012). The valence associated with life events tends to differ across age groups. In general, older adults tend to experience more developmental losses, which involve the decline or reduction of certain skills, resources, or opportunities that were previously available to them (Kaspar et al. 2023). Applied to the context of life events, this suggests that older adults might experience more negative life events, indicative of increasing developmental losses. This hypothesis underscores the notion that the experience of life events is shaped by the evolving developmental processes that occur throughout the lifespan (Diehl and Wahl 2010).

Life events, by their very nature, have the potential to disrupt the routine and equilibrium of individual’s everyday lives. As a result, they often introduce significant challenges and stressors, which can manifest as elevated levels of *strain* (Marum et al. 2014). It is crucial to note that increased strain is not solely a product of negative life events; even events characterized by overall positive valence, such as the birth of a child, can engender a notable degree of strain. Older adults might experience greater strain related to life events than their younger counterparts. As reported above, older adults tend to encounter a greater number of developmental losses; these losses can contribute to feelings of strain (Diehl and Wahl 2010; Kaspar et al. 2023). Moreover, life events that occur in later life may carry added complexities, such as dealing with health-related issues or navigating bereavement, which usually generate higher levels of strain (Lachman et al. 2011).

## Significant life events and well-being indicators

In this study, we adopt a multidimensional approach to operationalize well-being, encompassing subjective physical and mental health, subjective well-being, and life satisfaction, which are typical biopsychosocial indicators of well-being (e.g., Luhmann and Intelisano 2018). Life events often necessitate substantial behavioral adjustments in daily life, posing challenges to individual coping abilities (Thoits 2010) and overall well-being (Marum et al. 2014). As a result, the characteristics of life events can have consequences for health and well-being. Anticipation, age normativity, perceived control, valence, and strain of life events have all demonstrated associations with well-being in previous research. Specifically, life events that were more readily anticipated (Luhmann et al. 2012), were perceived as more age-normative (Shi and Brown 2021), more controllable (Lachman 2006; Lang and Heckhausen 2001), more positive (Gomez et al. 2009), and less strenuous (Marum et al. 2014) have been consistently linked with higher levels of well-being. In this study, we expect to replicate these findings.

In terms of age differences, older adults typically report higher subjective well-being and life satisfaction than younger adults (e.g., Charles and Carstensen 2010; Kunzmann et al. 2014; Mroczek and Spiro 2005), which is attributed to factors such as greater financial stability, longer-lasting marriages, and a greater focus on meaningful social relationships (Charles and Carstensen 2010; Marum et al. 2014).

In addition, developmental regulation theories propose that older adults are better able to cope with challenging life events compared to their younger counterparts: Older adults emphasize emotionally meaningful experiences (Carstensen 1992); they selectively process and recall more positive and less negative information (Kunzmann et al. 2014); and they apply secondary control (e.g., they alter their perception of the situation, adjust their goals, and seek meaning or positive reinterpretation; Heckhausen et al. 2019) and accommodation (e.g., they adapt their goals to the present circumstances; Brandtstädter 2009) in the face of difficulties.

Furthermore, older adults have gained life experiences resulting from personal and historical challenges that give older adults greater resilience in dealing with challenging situations (Cappeliez et al. 2008). Consequently, older adults experience comparable life events as overall less disruptive to their well-being than younger adults (Birditt et al. 2005; Gomez et al. 2009). Similarly, although older adults may face less controllable life events, they are more likely to exhibit a sense of mastery (Lachman 2006) and competence (Lachman et al. 2011).

Based on these findings, we expect that older adults, despite encountering life events that are less anticipated, less age-normative, less controllable, less positive, and more strenuous, will report higher levels of well-being than younger adults.

## The present study

This study examined age-related differences in the characteristics of significant life events and their age-differential association with well-being outcomes. Data were collected online from a large, age-diverse sample. Participants reported on one of 19 significant life events within the past two years, covering areas such as interpersonal relationships, work, health, or changes in place of residence. We formulated the following hypotheses:

1. Age is negatively associated with the anticipation, age normativity, perceived control, and valence of a life event, and positively associated with the strain of a life event. We also conducted an exploratory analysis of non-linear age effects.
2. Anticipation, age normativity, perceived control, and valence of a life event are positively related to subjective well-being, subjective physical and mental health, and life satisfaction, while strain is negatively related to these well-being indicators.
3. Age attenuates the associations hypothesized in H2, suggesting that the impact of life-event characteristics on well-being varies across adulthood.
4. To counteract the limitations inherent to a cross-sectional design, we considered the temporal distance between the survey (i.e., the time point of the assessment) and the occurrence of the life event in our analyses. Specifically, we hypothesized that the further back in time the life event occurred, the weaker the associations predicted in H2 (e.g., Nikitin et al. 2012). This expectation is based on the observation that the impact of significant life events on subjective well-being tends to diminish over time (Frederick and Loewenstein 1999).

## Methods

### Participants and procedure

The sample consisted of *N* = 6,688 participants (50.9% male, 48.9% female, 0.2% non-binary) with an age range of 18 to 90 years (*M* = 48.18, *SD* = 16.83). The participants were evenly distributed across the age decades (approx. 17% of participants per decade), except for participants over 70 years of age, who comprised 13.1% of the sample. The study included participants from Germany (78.9%), Austria (13.8%), and Switzerland (7%). A small number (0.2%) were German-speaking participants who reported a different country of residence. Among the participants, the highest level of education attained was vocational training (31.2%), followed by college/university (28.8%), high school (16.7%) and higher vocational school (15.4%). A smaller percentage (4.4%) had completed obligatory school, and 3.5% indicated “other” types of education.

The Ethics Committee of the University of Basel approved the data collection for this study (protocol number: 009–20–1). Participants were recruited through Respondi, an ISO-accredited recruitment agency, ensuring the inclusion of highly motivated participants through fair incentives and regular checks on identity and response behavior. Participants provided informed consent and were assured of data confidentiality. They received €3.60 for completing the 20 to 30-min online questionnaire. Data collection took place between June 23 and July 8, 2020. A screening questionnaire was used to invite participants who reported at least one significant life event from a prespecified list of 19 life events.

## Measures

### Significant life events

Participants read a list of 19 significant life events adapted from HILDA (Family, Income and Labour Dynamics in Australia; Wooden et al. 2002) and SHP (Swiss Household Panel; Voorpostel et al., 2016; see Supplementary Information Tables S1 and S2 for the list of individual life events and their frequencies in the sample). The participants selected the event that they had experienced in the two years prior to the study and that had the greatest impact on their lives. This approach should make the selection of the reported event comparable, ensuring consistency across participants. If none of the listed events were applicable, participants could select the “other” category and describe their life event. Most participants selected one of the 19 life events, indicating that the 19 life events represent a comprehensive selection of possible life events in adulthood. Participants who did not select any of the listed life events were not admitted to the further survey.

### Significant life-events characteristics

The results of the factor analyses suggested that anticipation and age normativity, as well as valence and strain, should be considered separate variables (for detailed results, see Supplementary Information S3).

**Anticipation.** Anticipation was operationalized by one self-developed item (i.e., “The event occurred unexpectedly”; 1 = *not at all* to 7 = *very*, reversed) based on the construct “expectation” (Frazier et al. 2011). Higher values indicate higher anticipation.

**Age normativity*.*** Age normativity was operationalized by one self-developed item based on the peer-shared experience (Leopold and Lechner 2015) of age-normative events (i.e., “Other people of my age also experience this or a similar event”; 1 = *not at all* to 7 = *very*). Higher values imply stronger age normativity.

**Perceived control*.*** Participants indicated how much control they had over the occurrence, process, and outcome of the life event (1 = *no control at all* to 7 = *full control*). The three items were aggregated to a scale representing overall perceived control (adapted from the Perceived Control Over Stressful Events scale; Frazier et al. 2011). The scale had a Cronbach’s α = 0.89. Higher scores indicate more perceived control.

**Valence***.* Participants rated the significant life event using the adjectives “positive”, “negative”, “desirable”, “undesirable”, “enriching”, and “burdening” (1 = *not at all* to 7 = *very*). Negatively formulated items were recoded, and all items were combined into one scale (Cronbach’s α = 0.97). The higher the score, the more positive and less negative a life event was experienced. These items were adapted from Luhmann et al. (2012) and the wording was slightly modified.

**Strain*.*** Participants rated the significant life event using the items “stressful” and “strenuous” (1 = *not at all* to 7 = *very*). The two items were aggregated to one scale (Cronbach’s α = 0.90). Higher scores indicate more strain. The items were adapted from Luhmann et al. (2012).

### Well-being indicators

**Subjective well-being**. Participants specified how they felt in the last 3–4 weeks (1 = *never* to 7 = *very often*), including 12 items such as “sleepy”, “alert”, “tense”, “calm” (Multidimensional Mood Questionnaire; Steyer et al. 1997). The items were recoded if necessary and aggregated to a scale (Cronbach’s α = 0.87). Higher scores indicate higher subjective well-being.

**Subjective physical and mental health.** Participants rated their physical and mental health, assessed by one item each: “All in all, how do you rate your physical/mental health?”; Idler and Benyamini 1997), with responses ranging from 1 = *very bad* to 7 = *excellent*. Higher scores indicate better physical/mental health.

**Life satisfaction.** Participants responded to questions of the Satisfaction with Life Scale (Diener et al. 1985). The answers to the five items ranged from 1 = *not true at all* to 7 = *very much true*. The items were recoded if applicable and aggregated to a highly reliable scale (Cronbach’s α = 0.91), with higher scores indicating higher life satisfaction.

### Age

Age was assessed in years and used as a continuous variable in the statistical analyses.

### Temporal distance

Participants indicated how long ago the life event ended, with response options ranging from *“more than two years ago”* to *“the event is still ongoing”* at monthly intervals, where higher scores correspond to the event ending further in the past.

For all items see Supplementary Information (Table S1).

## Data-analytical strategy

We conducted multilevel modeling,^1^ where specific life events were included at Level 2, with individuals nested within life events at Level 1. This approach allowed us to distinguish variance between different life events versus variance between different individuals experiencing the same life event. To investigate how age (linear, quadratic, and cubic) relates to life-event characteristics (H1), we conducted *across-event* comparisons (using multiple regression analysis) and *within-event* comparisons (using multilevel analysis and accounting for life events). By using both methodologies, we were able to distinguish between effects across life events, captured through conventional regression analysis (where age effects may partly reflect the effects of different life events experienced at different ages) and effects after accounting for the specific life event as captured through multilevel modeling. For all remaining analyses (H2–H4), we conducted only multi-level models. We report the fixed effects of predictors, random effects, and corresponding explained variances. In addition to random intercepts (i.e., life-event specific intercepts), we also tested for the presence of random slopes of the predictors (i.e., life-event specific slopes) by statistically comparing models with and without random slopes. A significantly better fit of a model with a random slope would indicate that the effect of a predictor depends on the life event in question. Concerning moderation analyses (H3 and H4), age and temporal distance were included simultaneously as moderators in the models. Both predictors and moderators were mean centered. The models are presented separately for each predictor and outcome combination. In addition, we aimed to control for gender and education. As including these two variables in the analyses resulted in negligible changes in the results without any systematic patterns, we omitted gender and education as control variables from the analyses. All analyses were conducted in R version 4.2.3 (R Core Team 2023), packages: “nlme” (v. 3.1–162, Pinheiro et al. 2023), “misty” (v. 0.4.11, Yanagida 2023), “reghelper” (v. 1.1.1, Hughes and Beiner 2022). The study was preregistered after establishing the hypotheses and before analysis (https://osf.io/cvkpr; see Supplementary Information, Table S4, for detailed deviations from the preregistration and their explanations).

## Results

### Descriptive analyses

The life events most frequently reported by participants were *loss of a close family member or a friend*, *own serious illness or injury,* and *birth of a child* (see Supplementary Information Table S2 for more information). For means, standard deviations, and correlations of all variables see Table 1. The mean values of the life-event characteristics were below the scale mid-point for anticipation, perceived control, and valence, and above the scale mid-point for age normativity, and strain. There were significant, but mostly weak to moderate correlations between the life event characteristics. Small to moderate negative correlations were found between age and all the characteristics (except for a positive correlation with strain), and positive correlations between age and all well-being indicators (except for a negative correlation with physical health). The mean temporal distance was 6 months since the end of the life event. Temporal distance was positively correlated with all well-being indicators.

**Table 1:** Means, standard deviations and correlations

Variable	*M*	*SD*	1	2	3	4	5	6	7	8	9	10	11
1. Age	48.18	16.83	(–)										
2. Temporal distance	34.06	9.04	0.03*	(–)									
3. Anticipation	3.71	2.41	−0.12**	0.10**	(0.30)								
4. Age normativity	4.98	1.75	−0.04**	0.03*	0.14**	(0.11)							
5. Perceived control	3.54	2.07	−0.18**	0.01	0.45**	0.11**	(0.47)						
6. Valence	3.63	2.36	−0.27**	−0.002	0.52**	0.16**	0.69**	(0.73)					
7. Strain	4.85	1.88	0.03*	0.06**	−0.25**	−0.11**	−0.31**	−0.52**	(0.30)				
8. Subjective well−being	4.63	1.12	0.18**	0.08**	0.09*	0.08**	0.17**	0.19**	−0.27**	(0.05)			
9. Subjective physical health	4.76	1.52	−0.27**	0.09**	0.14**	0.09**	0.21**	0.27**	−0.19**	0.42**	(0.11)		
10. Subjective mental health	4.87	1.56	0.05**	0.07**	0.12**	0.10**	0.18**	0.22**	−0.25**	0.71**	0.51**	(0.06)	
11. Life satisfaction	4.58	1.45	0.03*	0.05**	0.12*	0.10**	0.17**	0.22**	−0.20**	0.61**	0.46**	0.61**	(0.09)

*N* = 6,688, *p < 0.05. **p < 0.01. Elements on the diagonal (values in parentheses) are Intra-Class Correlation Coefficients (ICCs). The scale ranges of individual variables are as follows: for age (continuous, this sample: 18 to 90 years), for temporal distance (27 [still ongoing] to 53 [furthest in the past]), and for the remaining variables (1 to 7, with higher numbers indicating higher expression of the variable in question).The ICCs for characteristics of significant life events ranged from 0.11 (age normativity) to 0.73 (valence), which indicates that 11 to 73% of the variance were attributable to the specific life event, whereas the remaining variance was attributed to the individual who experienced the life event. It is important to note that some of the variance at Level 1, particularly for single-item variables such as anticipation and age normativity, may be attributable to measurement error. For well-being outcomes, ICCs ranged from 0.05 (subjective well-being) to 0.11 (physical health), indicating that the variance was mostly explained by individual factors rather than the specific life event. Multilevel modeling is recommended for ICCs of 0.05 and higher (Luo and Lai 2021)

H1: Age and characteristics of significant life events.

**Fig. 1:**
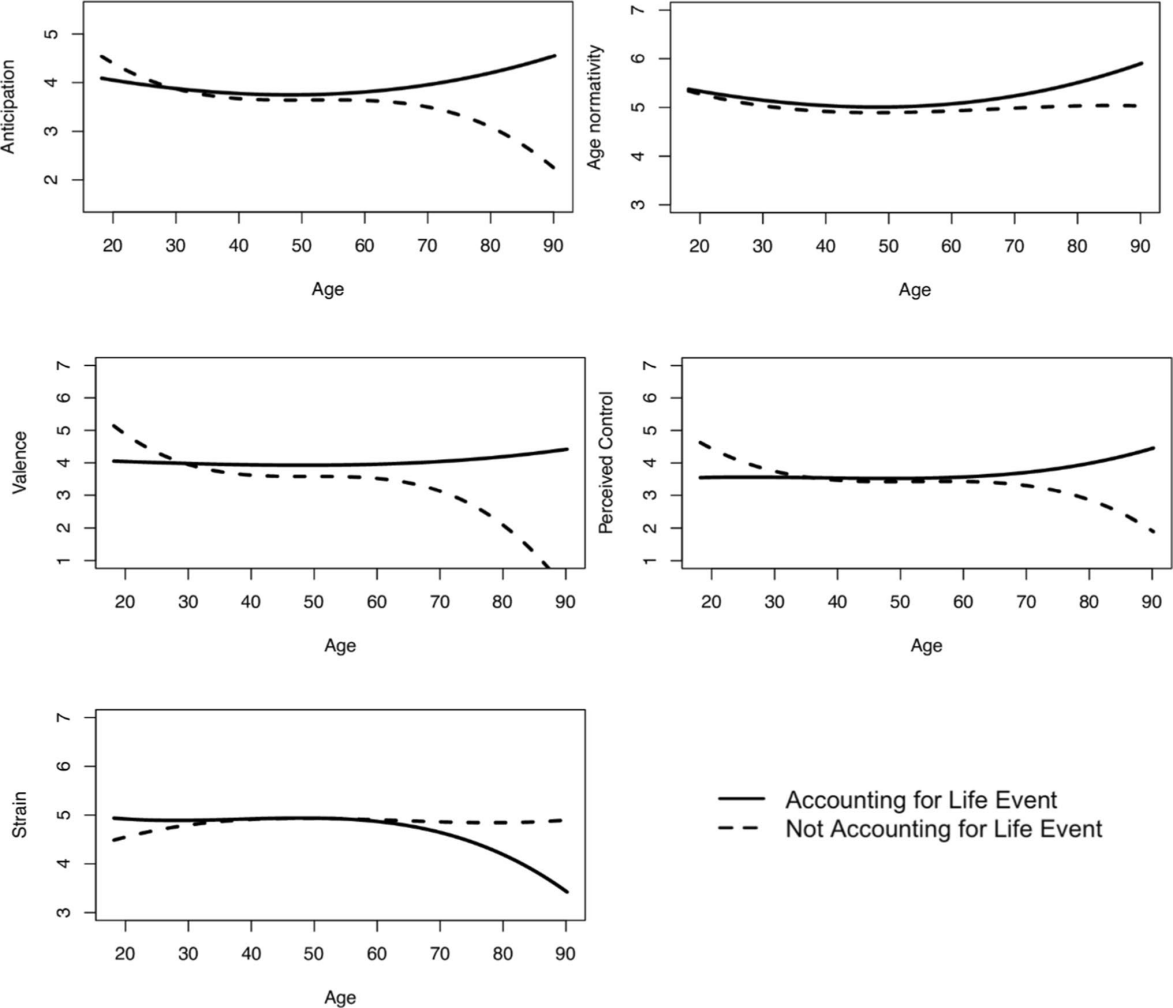
Age Effects on Characteristics of Significant Life Events. *Note.* Dashed lines represent mean age differences in life events, not accounting for the fact that individuals of different ages experienced different life events (multiple regression analyses). Solid lines represent age effects for individuals experiencing the same life event (multilevel analyses). For easier interpretation and comparison, we depicted an exemplary life event—the one with the life-event characteristic value closest to the overall mean (for anticipation: “other life event (open category)”; for age normativity: “unemployment”; for valence: “other life event (open category)”; for perceived control: “separation or divorce”; for strain: “relocation to another country”)

We hypothesized that age would be negatively associated with anticipation, age normativity, perceived control, and valence, and positively associated with strain of the life event. Consistent with our hypothesis, the multiple regression analyses revealed that when not accounting for the fact that different people experienced different life events, age was negatively associated with anticipation, perceived control, and valence (there were no significant linear associations with age normativity and strain, see Table 2, upper part). Additionally, we found a positive quadratic age effect on age normativity and perceived control and a negative quadratic age effect on strain. Finally, there was a positive cubic age effect on valence and perceived control. Figure 1 (dashed lines) illustrates these results: All characteristics of life events were most favorable in younger adulthood, relatively stable in middle age, and (with exception of age normativity and strain) declined in older adulthood. The age effects explained, however, relatively little variance (*R*^2^ ranging from 0.2 to 7.9%), indicative of negligible to small effect sizes (Cohen 1988).

**Table 2:** Linear, quadratic and cubic age effects on characteristics of significant life events

	Anticipation	Age normativity	Perceived control	Valence	Strain
*Not accounting for life event*					
Intercept	**3.651** (0.043)	**4.897** (0.031)	**3.442** (0.037)	**3.619** (0.041)	**4.933** (0.034)
Age	− **0.016** (0.004)	− 0.005 (0.003)	− **0.029** (0.003)	− **0.055** (0.004)	− 0.002 (0.003)
Age^2^	0.000 (0.000)	**0.000** (0.000)	**0.000** (0.000)	0.000 (0.000)	− **0.000** (0.000)
Age^3^	0.000 (0.000)	0.000 (0.000)	**0.000** (0.000)	**0.000** (0.000)	0.000 (0.000)
∆R^2^	0.013	0.003	0.035	0.079	0.002
*Accounting for life event*					
Fixed effects					
Intercept	**3.806** (0.298)	**4.757** (0.137)	**3.791** (0.317)	**3.908** (0.442)	**4.847** (0.246)
Age	**0.011** (0.004)	0.003 (0.003)	0.000 (0.003)	0.002 (0.002)	− **0.014** (0.003)
Age^2^	**0.000** (0.000)	**0.000** (0.000)	**0.000** (0.000)	**0.000** (0.000)	− **0.000** (0.000)
Age^3^	− **0.000** (0.000)	− 0.000 (0.000)	0.000 (0.000)	− 0.000 (0.000)	0.000 (0.000)
*Random effects*					
Intercept	1.746 (2.5%)	0.351 (–)	1.989 (2.6%)	3.898 (–)	1.187 (5.2%)
Residual	4.034 (–)	2.734 (–)	2.203 (–)	1.418 (–)	2.543 (1.2%)

Two models: not accounting for life event (multiple regression analysis) and accounting for life event (multilevel analysis).

Age was mean centered. Fixed effects: Standard errors in parentheses. Significant effects in bold. Random effects: ∆R^2^ in parentheses. – = < 1% ∆R^2^

In comparison to the multiple regressions analysis, the results of the multilevel fixed-effect analyses with random intercepts showed a different picture of the effects of age on the life-events characteristics (see Table 2, lower part): Linear age was positively associated with anticipation and negatively associated with strain. Quadratic age was positively associated with anticipation, age normativity, perceived control, and valence, and negatively with strain.

Finally, there was a negative cubic age effect on anticipation. Figure 1 (solid lines) illustrates these results for exemplary life events (always the one life event closest to the mean value of the respective characteristic): In opposite to the results of the multiple regression analysis, all characteristics of the life events were *most* favorable in older adulthood and relatively stable in young adulthood and middle age (with exception of anticipation and age normativity that showed a U-shape). In other words: In terms of life-event characteristics, older adults experienced less favorable life events but experienced the same life event more favorably than younger and (with respect to anticipation and age normativity) particularly middle-aged adults. However, age explained between less than 1% and up to 5.2% of variance in the life-event characteristic, indicating negligible to small effect sizes (Cohen 1988).

In addition to random intercepts, which represent the average effects of age across life events, we also examined random slopes of age. Random slopes indicate life event-specific effects of age. Models with random slopes showed an improved model fit for all life event characteristics, indicating that the effect of age on anticipation, age normativity, perceived control, valence, and strain depended on the life event under consideration. For example, older adults did not perceive all life events as more normative than younger adults, but only specific life events, whereas other life events were perceived to be more normative by younger adults. For the comparisons of models without and with random slopes, see Supplementary Information Table S5.

H2: Characteristics of significant life events and well-being indicators.

Regarding well-being indicators, we hypothesized that anticipation, age normativity, valence, and perceived control of a life event would be positively linked to the well-being indicators, whereas strain would be negatively linked to the well-being indicators. The fixed-effect results supported the hypothesis for age normativity, valence, perceived control, and strain. Contrary to our hypothesis, anticipation was negatively associated with subjective well-being and showed no significant association with any other well-being indicator (see Table 3).

**Table 3:** Multilevel models of fixed and random effects for significant life event characteristics on well-being outcomes

	Subjective well-being	Physical health	Mental health	Life satisfaction
*Fixed effects*				
Intercept	**4.562** (0.102)	**4.426** (0.140)	**4.510** (0.138)	**3.938** (0.141)
Anticipation	− **0.015** (0.007)	− 0.013 (0.009)	− 0.001 (0.009)	− 0.012 (0.009)
Age normativity	**0.030** (0.008)	**0.022** (0.010)	**0.050** (0.011)	**0.039** (0.010)
Control	**0.086** (0.010)	**0.085** (0.013)	**0.096** (0.013)	**0.104** (0.012)
Valence	**0.064** (0.013)	**0.075** (0.017)	**0.102** (0.018)	**0.112** (0.016)
Strain	− **0.128** (0.009)	− **0.057** (0.012)	− **0.143** (0.012)	− **0.082** (0.011)
*Random effects*				
Intercept	0.070 (–)	0.150 (41%)	0.109 (30%)	0.162 (19.8%)
Residual	1.119 (8%)	1.928 (2.5%)	2.173 (6.4%)	1.848 (5.1%)

Fixed effects: Standard errors in parentheses. Significant effects in bold (*p* < 0.05). Random effects: ∆R^2^ in parentheses

In terms of effect sizes, life-event characteristics explained less than 1% of the variance in subjective well-being at the level of life events and a small proportion of the variance (8%) at the individual level. For all other indicators of well-being, life-event characteristics explained a substantial proportion of the variance at the life-event level (up to 41%), whereas at the individual level they explained only a small proportion of the variance (up to 6%; see Table 3). These results indicate that differences in well-being indicators (except for subjective well-being) attributable to life-event characteristics were more strongly associated with specific life events than with specific individuals.

In addition, we identified improved model fits of models with random slopes for 12 out of the 20 models (see Supplementary Information Table S6), suggesting that the relationships between life-event characteristics and well-being outcomes differ between life events.

H3 & H4: Age and temporal distance as moderators.

**Fig. 2:**
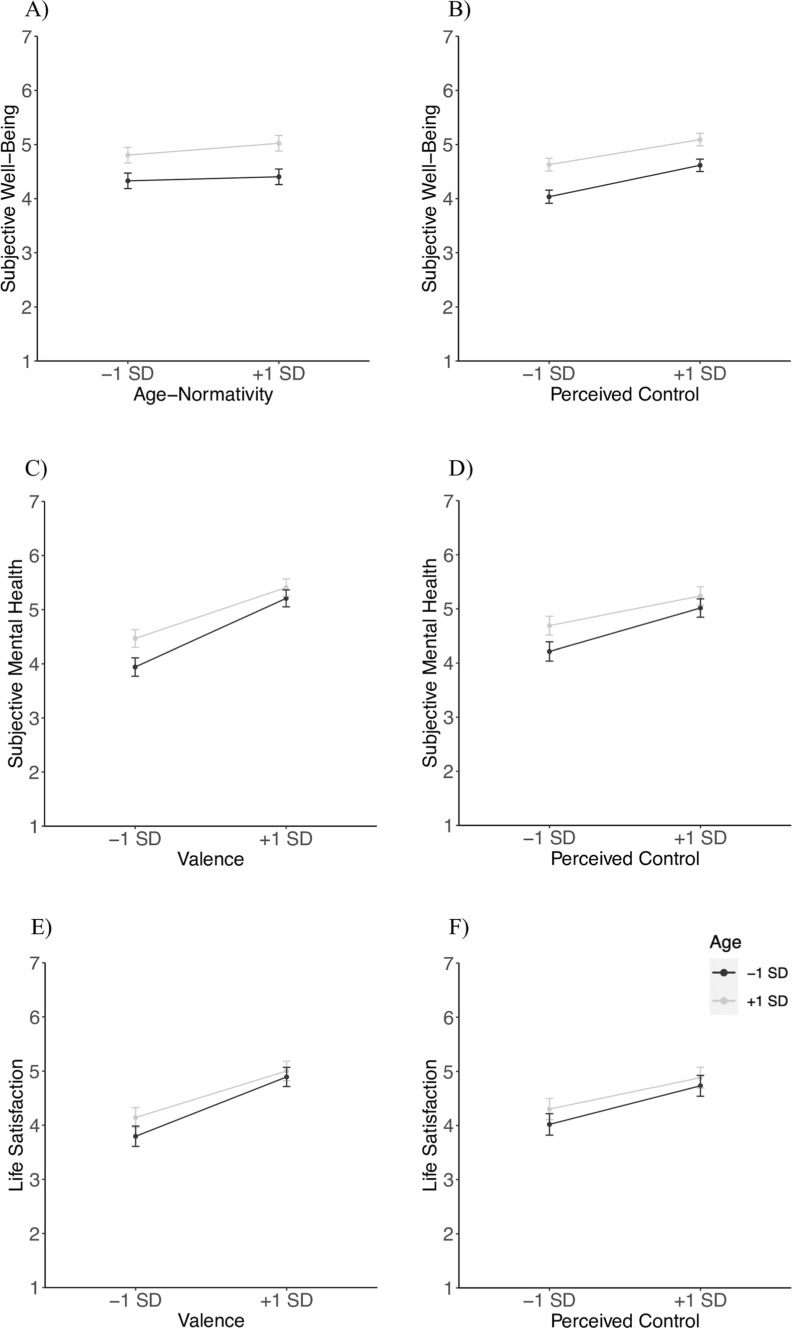
Significant Multilevel Age Interactions Between Characteristics and Well-Being Outcomes. *Note.* The black line represents -1 SD of age (31 years), and the grey line + 1 SD of age (65 years)

Finally, we tested our hypothesis that age and temporal distance act as moderators of the relationships postulated in H2. The moderation models were conducted separately for each predictor and outcome combination. When considering age as a moderator, the fixed-effect results supported our hypothesis for the relationship between valence and mental health, valence and life satisfaction, and perceived control and life satisfaction (see Fig. 2 A–F). These relationships were indeed weaker with higher age, which aligns with our hypothesis. Contrary to our hypothesis, we found a stronger relationship between age normativity and subjective well-being with higher age. All other results spoke in favor of age-independent relations between life event characteristics and well-being.

With temporal distance as a moderator, the fixed effects revealed significant interactions in all cases except for age normativity and perceived control as predictors of life satisfaction, and perceived control as predictor of mental health. More variance was again explained at the life-event level (up to 60%), whereas the proportion of explained variance at the individual level varied from 1 to 11% (see Supplementary Information Table S7). Again, the results were mostly in favor of our hypothesis that a larger temporal distance weakens the association between life-event characteristics and well-being.

Finally, we found that all models fit better when allowing for random slopes (i.e., life-event specific effects) of age. Random slopes of temporal distance improved model fit in 11 out of 20 models, indicating that for some outcomes (particularly anticipation and age normativity) the effects of temporal distance were the same across life events, while for other outcomes they were life-event specific. For more detailed results see Supplementary Information Table S8.

## Discussion

The purpose of this study was to identify characteristics of life events that vary with age and to examine how age moderates the associations between characteristics and indicators of well-being. In summary, we found that (1) although older compared to younger adults experienced life events that were more challenging (in terms of life-event characteristics), they experienced the same life event as less challenging. Furthermore, (2) well-being indicators were similarly associated with life-event characteristics regardless of age. Finally, (3) time attenuated the associations between life-event characteristics and well-being indicators, supporting the validity of the study design. In the following, we discuss these findings and their implications.

## Life-event characteristics across the lifespan

The results of the present study supported our first hypothesis that life-event characteristics are less favorable in older compared to younger adulthood, but only when not accounting for the specific live event experienced: Older adults reported life events that were less anticipated, less normative, less controllable, lower in valence, and more strenuous. The trajectories were slightly different for the different characteristics (sometimes being stable from middle age onwards), but the direction of the effects was always towards less favorable characteristics with increasing age. However, when accounting for differences in characteristics between life events, the results showed that anticipation, age normativity, perceived control, and valence were higher, and strain was lower in older adulthood compared to younger adults. That means that older adults are generally more likely to experience highly challenging life events, such as the death of a loved one, which could raise their overall ratings of life event challenges. However, when encountering a specific event such as a family member’s death, older adults may perceive it as less overwhelming or negative compared to younger adults.

Interestingly, however, it was rather middle adulthood that was associated with more challenging characteristics (particularly in terms of anticipation and age normativity). This might be the case because middle adulthood is the most intense and demanding phase of life (Mehta et al. 2020) and coping with life events during this time might add to the already high demands. However, more favorable experience of life events in older adulthood could also be due to factors such as wisdom gained from life experience, higher resilience, or a shift in priorities and perspectives in older adults (Cappeliez et al. 2008; Carstensen 1992). In general, though, age explained little variance in life event characteristics. Across a wide range of life events, we found that adults of different ages experience more similarities than differences in life-events characteristics. Nevertheless, our results call for further investigation of age differences across and within life events.

## Life-event characteristics and well-being across the lifespan

Our results suggest that individuals who reported higher age normativity, higher perceived control, more positive valence, and less strain in the context of their life events also reported higher levels of subjective well-being, subjective mental and physical health, and life satisfaction. Perceived control, valence, and strain yielded the most robust findings, and explained the most variance, particularly in the health outcomes. These results mostly confirm our hypotheses (except for anticipation, which was negatively associated with subjective well-being) and are in line with previous literature (Lachman et al. 2011; Leopold and Lechner 2015; Wrosch and Freund 2001). The reason for anticipation yielding unexpected results may be that, unlike the other four constructs, it can be characterized by ambivalence. It can encompass both a period of stress and expected challenges, and a time of hope, promise, and a joyful anticipation. The nature of anticipation encompasses this dualism, where individuals may experience a mix of positive and negative emotions as they anticipate a future event or outcome (e.g., birth of a child; Rilby et al. 2012). In general, life-event characteristics (except for subjective well-being) explained more variance in the well-being indicators at the life-event level, suggesting that the experience of a significant life event is more common rather than unique (see a meta-analysis focusing on general patterns and trends in how people react and adapt to life events; Luhmann et al. 2012).

Our hypothesis that age moderates the relationship between life-event characteristics and well-being was not supported. Most age moderations were weak and did not reach significance. This suggests that the relation between life event characteristics and well-being is comparable across the adult age range. Our results on the age effects on life-event characteristics suggest that the coping mechanisms might occur earlier, when older adults evaluate the life event in terms of its characteristics. These findings are consistent with the idea that older adults achieve well-being by selecting and optimizing situations and information before they can have negative impact on their well-being (Urry and Gross 2010). However, when negative experiences occur, older adults may not have an advantage in terms of their emotional reaction (Charles and Carstensen 2010).

Still, there were some results in line with our hypotheses and previous literature on better emotion regulation in older adults (Kunzmann et al. 2014). The associations between perceived control and valence on the one side and mental health and life satisfaction on the other side were weaker among older compared to younger adults. This implies that—to some degree—older adults coped better with less positive and less controllable life events (see Nikitin et al. 2022, for a similar conclusion). Literature suggests that primary control diminishes with age, while secondary control increases (Brandtstädter 2009; Heckhausen et al. 2019). Adults may hence shift their coping strategies from primary control (actively influencing life events) to secondary control (greater acceptance, emotional regulation, and reframing of life events) as they age. This shift in coping strategies may contribute to promoting mental health and life satisfaction in later life, even in the face of various challenges and stressors. Also, our results showed that age normativity was more positively associated with subjective well-being with increasing age, which could be explained by a higher importance of adhering to age-related norms in today’s older generations and the weakening of age-norms importance for today’s younger generations, as suggested by Liefbroer and Billari (2010), Bühler and Nikitin (2020), or Shi and Brown (2021).

Moreover, the analysis showed that age does not moderate well-being in the same way for all events, but dependent on the type of the life event. In other words, certain life events may exhibit a stronger age-related effect on well-being, while others may show a weaker or negligible age-related effect (Brose et al. 2013). While certain effects were consistent across different life events, each specific life event also brought its own unique circumstances and consequences, resulting in different life event-specific effects. Future studies are needed to more systematically investigate when and why this is the case.

## The role of temporal distance

Our findings suggest that temporal distance acted as a consistent moderator between life-event characteristics and well-being outcomes. The associations between life-event characteristics and indicators of well-being were weaker with increasing time since the event. This finding aligns with the notion of hedonic adaptation, suggesting that individuals eventually adapt to the effects of life events on their well-being, returning to their baseline level of happiness (Luhmann and Intelisano 2018).

This suggests that the effects of life-event characteristics on well-being are not static but diminish over time (Frederick and Loewenstein 1999). Although cross-sectional studies have their limitations in capturing these changes, recognizing the importance of temporal distance as a moderator in the relationship between life-event characteristics and well-being outcomes, as was done in this study, is critical to gaining greater insight into how people adapt to life events over time.

## Limitations and implications for future research

The strengths of the present study are the large sample, a high number of different significant life events represented across participants, and the consideration of various life-event characteristics and well-being indicators. However, our study also has some limitations. First, causality cannot be established. Furthermore, the observed age differences could be an expression of cohort differences (e.g., differences in socialization and experiences) rather than aging processes (Drewelies et al. 2019). Also, age effects are likely to be at least partly explained by proximal variables such as health, resilience, or life experience. Future research should address these as potential covariates. Additionally, some variables were measured using only a single-item scale, raising questions about the extent to which they fully capture the underlying construct, such as the complexity of age normativity. Although we used temporal distance as a moderator to validate the cross-sectional data, longitudinal research is needed to better understand the relationships and their underlying mechanisms. Moreover, recruiting participants online offers clear advantages by facilitating rapid data collection from large samples but may introduce selection bias, particularly regarding older adults, despite the increasing use of technology by older adults (Faverio 2022). Data assessment during the COVID-19 pandemic could influence the prevalence and characteristics of certain life events and their characteristics. Similarly, the fact that life events were assessed retrospectively is a potential limitation, as it may lead to recall biases when responding to or interpreting the questions. Finally, our study involved numerous analyses, increasing the risk of Type I errors. Given our large sample size, however, we chose not to apply alpha-level corrections, as such procedures tend to increase the likelihood of Type II errors (Nakagawa 2004). Additionally, alpha corrections often place excessive emphasis on significance levels rather than on effect sizes and explained variance, which we prioritize in our study. We believe that these indicators provide a more accurate measure of meaningful results.

## Conclusion

Our findings suggest that while life events can become more challenging with age, the impact of these events in terms of their characteristics may diminish or even reverse as people grow older. Overall, the similarities in how people of different ages experience major life events outweigh the differences, highlighting that challenging times across adulthood share more commonalities than distinctions.

## Supplementary Information

Below is the link to the electronic supplementary material.

Supplementary file1 (DOCX 76 KB)

## Data Availability

No datasets were generated or analysed during the current study. The current study was preregistered. Material and analysis code are available at OSF (https://osf.io/cvkpr). Data is available upon request.

## Footnotes

1 We decided to use multilevel analyses instead of the preregistered analyses because this allows for a more fine-grained and comprehensive investigation of life-event specific mean values and predictor effects.
