# Supplementary material for: Navigating life’s twists and turns: characteristics of life events across adulthood
Source: Eur J Ageing. 2025 Jan 20;22(1):2. doi: 10.1007/s10433-025-00838-0 (PMC11743415; doi:10.1007/s10433-025-00838-0)
Supplement: Supplementary file 1 — Supplementary file1 (DOCX 76 KB) [file 10433_2025_838_MOESM1_ESM.docx]

**Supplementary Information**

**Table S1**

German and English Wording of the Items

|  | German Item | English Translation | Subscale |
| --- | --- | --- | --- |
| 1 | Umzug in ein anderes Land | Relocation to another country | Life Event |
| 2 | Umzug in eine andere Stadt/einen anderen Wohnort | Relocation to another city / place of residence | Life Event |
| 3 | Wohnungswechsel am gleichen Wohnort | Change of residence in the same city | Life Event |
| 4 | Längerer Aufenthalt im Ausland | Longer stay abroad | Life Event |
| 5 | Heirat/ eingetragene Partnerschaft | Marriage / registered partnership | Life Event |
| 6 | Eine neue romantische Beziehung eingegangen (von mindestens einem Monat Dauer) | Entered a new romantic relationship (of at least a month) | Life Event |
| 20 | Geburt eines Kindes | Birth of a child | Life Event |
| 7 | Trennung oder Scheidung | Separation or divorce | Life Event |
| 8 | Eine wichtige soziale Beziehung beendet (ausser Trennung oder Scheidung) | End of an important social relationship (other than separation or divorce) | Life Event |
| 9 | Eigene schwerwiegende Krankheit oder Verletzung | Own serious illness or injury | Life Event |
| 10 | Schwerwiegende Krankheit oder Verletzung des Partners/der Partnerin | Serious illness or injury of the partner | Life Event |
| 11 | Schwerwiegende Krankheit oder Verletzung eines nahen Familienmitgliedes oder Freundes/Freundin | Serious illness or injury of a close family member or a friend | Life Event |
| 12 | Tod des Partners/der Partnerin | Loss of the partner | Life Event |
| 13 | Tod eines nahen Familienmitgliedes oder Freundes/Freundin | Loss of a close family member or a friend | Life Event |
| 14 | Längerer Aufenthalt im Spital/Psychiatrie/Gefängnis oder vergleichbarer Institution | Long stay in hospital / psychiatry / prison or comparable institution | Life Event |
| 15 | Beförderung im Job | Job promotion | Life Event |
| 16 | Stellenwechsel | Job change | Life Event |
| 17 | Arbeitslosigkeit | Unemployment | Life Event |
| 18 | Verrentung | Retirement | Life Event |
| 1.  2.  3.  26.  27. | Geben Sie bitte an, wann das Ereignis beendet war.  Das Ereignis dauert noch an.  vor weniger als einem Monat  vor 1 Monat  …  vor 24 Monaten  vor mehr als 24 Monaten | Please indicate when the event ended.  The event is still ongoing.  less than a month ago  a month ago  …  24 months ago  more than 24 months ago | Temporal Distance  Temporal Distance  Temporal Distance  Temporal Distance  Temporal Distance  Temporal Distance |
| 1.  2. | Wie stark war das Ereignis vorhersehbar?  Das Ereignis trat unerwartet ein.  Andere Menschen in meinem Alter erleben dieses oder ähnliche Ereignisse auch. | How predictable was the event?  The event occurred unexpectedly.  Other people my age also experience this or similar events. | Anticipation (R)  Age-normativity |
|  | Wie viel Kontrolle hatten Sie über die verschiedenen Phasen des Ereignisses? | How much control did you have over the different stages of the event? |  |
| 1. | Wie viel Kontrolle hatten Sie über das Eintreten des Ereignisses? | How much control did you have over the occurrence of the event? | Perceived Control |
| 2.  3. | Wie viel Kontrolle hatten Sie über den Verlauf des Ereignisses?  Wie viel Kontrolle hatten Sie über den Ausgang des Ereignisses? | How much control did you have over the course of the event?  How much control did you have over the outcome of the event? | Perceived Control  Perceived Control |
| 1.  2.  3.  4.  5.  6.  7.  8. | Wie haben Sie das Ereignis erlebt?  Positiv  Negativ  Erwünscht  Unerwünscht  Belastend  Bereichernd  Anstrengend  Stressig | How did you experience the event?  Positive  Negative  Desirable  Undesirable  Burdening  Enriching  Strenuous  Stressful | Valence  Valence  Valence  Valence  Valence  Valence  Strain Strain |
| 1.  2.  3.  4.  5.  6.  7.  8.  9.  10.  11.  12. | Im Folgenden finden Sie eine Liste von Wörtern, die verschiedene Stimmungen beschreiben. Bitte gehen Sie die Wörter der Liste nacheinander durch und klicken Sie jeweils die Antwort an, die am besten beschreibt, wie häufig Sie sich in den vergangenen Wochen so gefühlt haben.  Wie häufig haben Sie sich in den vergangenen 3-4 Wochen so gefühlt?  schläfrig  wohl  ausgeglichen  unglücklich  wach  unzufrieden  angespannt  frisch  glücklich  nervös  ermattet  ruhig | Below is a list of words that describes different moods. Please go through the list of words one by one and click on the answer that best describes how often you have felt this way in the past few weeks.  How often have you felt this way in the past 3-4 weeks?  sleepy  good  at ease  unhappy  alert  discontent  tense  fresh  happy  nervous  exhausted  calm | SWB (R)  SWB  SWB  SWB (R)  SWB  SWB (R)  SWB (R)  SWB  SWB  SWB (R)  SWB (R)  SWB |
|  | Alles in allem, wie schätzen Sie Ihre körperliche Gesundheit ein?  Alles in allem, wie schätzen Sie Ihre seelische Gesundheit ein? | All in all, how would you rate your physical health?  All in all, how would you rate your mental health? | Physical Health  Mental Health |
| 1.  2.  3.  4.  5. | Nachfolgend finden Sie fünf Aussagen, denen Sie zustimmen oder nicht zustimmen können. Nutzen Sie die Antwortskala, um das Ausmaß Ihrer Zustimmung anzugeben.  In den meisten Bereichen entspricht mein Leben meinen Idealvorstellungen.  Meine Lebensbedingungen sind ausgezeichnet.  Ich bin mit meinem Leben zufrieden.  Bisher habe ich die wesentlichen Dinge erreicht, die ich mir für mein Leben wünsche.  Wenn ich mein Leben noch einmal leben könnte, würde ich kaum etwas ändern. | Below are five statements that you can agree or disagree with. Use the response scale to indicate your level of agreement.  In most ways my life is close to my ideal.  The conditions of my life are excellent.  I am satisfied with my life.  So far, I have gotten the important things I want in life.  If I could live my life over, I would change almost nothing. | Life Satisfaction  Life Satisfaction  Life Satisfaction  Life Satisfaction  Life Satisfaction |
| *Note.* SWB = Subjective Well-Being. | | | |

Table S2

Individual Significant Life Events and Their Descriptive Statistics

| Significant Life Event | N | % of the sample | M  (age) | Correlation with age |
| --- | --- | --- | --- | --- |
| Relocation to another country | 121 | 1.81 | 38.12 | -.08** |
| Relocation to another city / place of residence | 466 | 6.97 | 39.20 | -.15** |
| Change of residence in the same city | 281 | 4.20 | 45.23 | -.04** |
| Longer stay abroad | 203 | 3.04 | 45.76 | -.03* |
| Marriage / registered partnership | 233 | 3.48 | 44.23 | -.05** |
| Entered a new romantic relationship (of at least a month) | 374 | 5.59 | 38.17 | -.15** |
| Birth of a child | 585 | 8.75 | 39.21 | -.17** |
| Separation or divorce | 302 | 4.52 | 43.36 | -.06** |
| End of an important social relationship (not divorce) | 186 | 2.78 | 44.85 | -.03** |
| Own serious illness or injury | 834 | 12.47 | 58.05 | .22** |
| Serious illness or injury of the partner | 315 | 4.71 | 59.52 | .15** |
| Serious illness or injury of a close family member or a friend | 390 | 5.83 | 49.78 | .02 |
| Loss of the partner | 136 | 2.03 | 64.81 | .14** |
| Loss of a close family member or a friend | 1054 | 15.76 | 52.30 | .11** |
| Long stay in hospital / psychiatry / prison (or other institution) | 71 | 1.06 | 48.44 | .002 |
| Job promotion | 156 | 2.33 | 39.82 | -.08** |
| Job change | 360 | 5.38 | 40.76 | -.11** |
| Unemployment | 248 | 3.71 | 45.72 | -.03* |
| Retirement | 253 | 3.78 | 62.89 | .17** |
| Other life event | 120 | 1.79 | 52.30 | .03** |

*Note. N* = 6,688, **p* < .05. ***p* < .01.

S3

To determine whether the variables anticipation and age normativity (one item each) should be combined to one single variable or remain separate, we analyzed their bivariate correlation. The results revealed a small correlation (r = .14), indicating two separate constructs. Similarly, to determine whether the initial eight items depicting valence (the ones above plus “strenuous” and “stressful”) should be one single construct or more, we also conducted a factor analysis. The results of a one-factor model showed an insufficient fit to the data (RMSEA = .286, TLI = .758, BIC = 10759.05). A two-factor solution fit the data better (RMSEA = .152, TLI = .931, BIC = 1910.04), suggesting that valence and strain should be considered separate. The bivariate correlation for valence and strain revealed a moderate (negative) correlation (r = -.52).

Table S4

Deviations From the Preregistration

| Preregistration | Changes in Manuscript |
| --- | --- |
| Age normativity was considered to contain two items (including the item on anticipation). | After conducting factor analysis, we determined that age normativity and anticipation were distinct constructs. |
| We originally intended to perform multiple regression analyses. | We decided that using multilevel modeling provides more detailed results and interpretations, allowing us to explore both between- and within-life event variations. |
| We planned to analyze individual life events and conduct structural equation modeling as part of our exploratory analysis. | With the implementation of multilevel modeling, individual life event analyses were no longer unnecessary. |

| Table S5 |  | |  |  | |  |  |
| --- | --- | --- | --- | --- | --- | --- | --- |
| ANOVA Comparison of Models with and without Random Slopes for Age | | | | | | | |
|  | AIC | BIC | | | Log-Likelihood | p | |
| Anticipation | 28443.96 | 28471.19 | | | -14217.98 |  | |
| Random Slope Age | 28415.71 | 28456.55 | | | -14201.85 | <.001 | |
| Age-Normativity | 25828.33 | 25855.57 | | | -12910.17 |  | |
| Random Slope Age | 25553.09 | 25593.93 | | | -14201.85 | <.001 | |
| Perceived Control | 24401.62 | 24428.85 | | | -12196.81 |  | |
| Random Slope Age | 24363.71 | 24404.56 | | | -12175.85 | <.001 | |
| Valence | 21478.15 | 21505.38 | | | -10735.08 |  | |
| Random Slope Age | 21452.99 | 21493.84 | | | -10720.50 | <.001 | |
| Strain | 25364.52 | 25391.75 | | | -12678.26 |  | |
| Random Slope Age | 25310.08 | 25350.93 | | | -12649.04 | <.001 | |

*Note.* Degrees of freedom = 4, 6. AIC = Akakike information criterion; BIC = Bayesian information criterion.

| Table S6 |  | |  |  | |  |  |
| --- | --- | --- | --- | --- | --- | --- | --- |
| ANOVA Comparison of Models with and without Random Slopes | | | | | | | |
|  | AIC | BIC | | | Log-Likelihood | p | |
| Subjective Well-Being | 19840.45 | 19894.91 | | | -9912.226 |  | |
| Random Slope |  |  | | |  |  | |
| Anticipation | 19840.86 | 19908.93 | | | -9910.429 | .17 | |
| Age-Normativity | 19825.48 | 19893.55 | | | -9902.741 | <.001 | |
| Control | 19820.94 | 19889.01 | | | -9900.470 | <.001 | |
| Valence | 19827.25 | 19895.32 | | | -9903.623 | <.001 | |
| Strain | --- | --- | | | --- | --- | |
| Physical Health | 23482.53 | 23536.99 | | | -11733.27 |  | |
| Random Slope |  |  | | |  |  | |
| Anticipation | 23483.65 | 23551.72 | | | -11731.82 | .24 | |
| Age-Normativity | 23474.11 | 23542.18 | | | -11727.05 | .002 | |
| Control | 23439.72 | 23507.79 | | | -11709.86 | <.001 | |
| Valence | 23451.19 | 23519.26 | | | -11715.60 | <.001 | |
| Strain | 23460.97 | 23529.04 | | | -11720.49 | <.001 | |
| Mental Health | 24272.24 | 24326.69 | | | -12128.12 |  | |
| Random Slope |  |  | | |  |  | |
| Anticipation | 24272.58 | 24340.66 | | | -12126.29 | .16 | |
| Age-Normativity | 24273.51 | 24341.58 | | | -12126.75 | .26 | |
| Control | 24260.61 | 24328.68 | | | -12120.31 | <.001 | |
| Valence | 24266.18 | 24334.25 | | | -12123.09 | .007 | |
| Strain | 24276.02 | 24344.09 | | | -12128.01 | .90 | |
| Life Satisfaction | 23200.22 | 23254.68 | | | -11592.11 |  | |
| Random Slope |  |  | | |  |  | |
| Anticipation | 23192.22 | 23260.29 | | | -11586.11 | .003 | |
| Age-Normativity | 23203.29 | 23271.37 | | | -11591.65 | .63 | |
| Control | 23176.92 | 23244.99 | | | -11578.46 | <.001 | |
| Valence | 23187.81 | 23255.88 | | | -11583.90 | <.001 | |
| Strain | --- | --- | | | --- | --- | |
| *Note.* Degrees of freedom = 20, 29. AIC = Akakike information criterion; BIC = Bayesian information criterion; -- = no fit. | | | | | | | |

| Table S7 |  |  |  | |  |  |  |  |
| --- | --- | --- | --- | --- | --- | --- | --- | --- |
| Multilevel Model of Fixed and Random Effects for Significant Life Event Characteristics on Well-Being Outcomes Moderated by Age and Temporal Distance | | | | | | | | |
|  | Subjective  Well-Being | Physical Health | | Mental Health | | Life Satisfaction | |  |
| Fixed Effects | **4.628** (0.060) | **4.838** (0.115) | | **4.845** (0.091) | | **4.536** (0.103) | |  |
| Intercept | **4.634** (0.068) | **4.828** (0.093) | | **4.842** (0.097) | | **4.535** (0.102) | |  |
| Age | **0.016** (0.001) | **-0.017** (0.001) | | **0.010** (0.001) | | **0.007** (0.001) | |  |
| Temporal Distance | **0.010** (0.002) | **0.011** (0.002) | | **0.013** (0.002) | | **0.006** (0.002) | |  |
| Anticipation | **0.024** (0.006) | **0.024** (0.009) | | **0.050** (0.009) | | **0.036** (0.008) | |  |
| Anticipation*Age | -0.000 (0.000) | -0.000 (0.000) | | -0.001 (0.000) | | -0.000 (0.000) | |  |
| Anticipation*Temp | **-0.002** (0.001) | **-0.002** (0.001) | | **-0.003** (0.001) | | **-0.002** (0.001) | |  |
| Random Effects |  |  | |  | |  | |  |
| Intercept | 0.088 (31%) | 0.164 (36%) | | 0.176 (12.8%) | | 0.198 (2%) | |  |
| Residual | 1.147 (5.8%) | 1.908 (3.5%) | | 2.273 (2.2%) | | 1.931 (1%) | |  |
|  |  |  | |  | |  | |  |
|  |  |  | |  | |  | |  |
| Fixed Effects | **4.628** (0.060) | **4.838** (0.115) | | **4.845** (0.091) | | **4.536** (0.103) | |  |
| Intercept | **4.640** (0.071) | **4.832** (0.095) | | **4.855** (0.101) | | **4.544** (0.105) | |  |
| Age | **0.016** (0.001) | **-0.017** (0.001) | | 0.010 (0.001) | | **0.007** (0.001) | |  |
| Temporal Distance | **0.010** (0.002) | **0.011** (0.002) | | **0.013** (0.002) | | **0.006** (0.003) | |  |
| Normativity | **0.042** (0.008) | **0.036** (0.010) | | **0.068** (0.011) | | **0.055** (0.010) | |  |
| Normativity*Age | **0.001** (0.000) | -0.000 (0.001) | | 0.001 (0.001) | | -0.000 (0.001) | |  |
| Normativity*Temp | **-0.002** (0.001) | **-0.003** (0.001) | | -0.002 (0.001) | | -0.002 (0.001) | |  |
| Random Effects |  |  | |  | |  | |  |
| Intercept | 0.096 (43%) | 0.171 (33%) | | 0.196 (25.6%) | | 0.211 (4.5%) | |  |
| Residual | 1.145 (5.7%) | 1.907 (3.5%) | | 2.274 (2.2%) | | 1.929 (1.1%) | |  |
|  |  |  | |  | |  | |  |
|  |  |  | |  | |  | |  |
| Fixed Effects | **4.628** (0.060) | **4.838** (0.115) | | **4.845** (0.091) | | **4.536** (0.103) | |  |
| Intercept | **4.594** (0.056) | **4.789** (0.084) | | **4.790** (0.083) | | **4.485** (0.094) | |  |
| Age | **0.016** (0.001) | **-0.017** (0.001) | | **0.010** (0.001) | | **0.006** (0.001) | |  |
| Temporal Distance | **0.009** (0.002) | **0.010** (0.002) | | **0.012** (0.002) | | **0.005** (0.002) | |  |
| Control | **0.126** (0.009) | **0.124** (0.011) | | **0.163** (0.012) | | **0.156** (0.011) | |  |
| Control*Age | -0.001 (0.000) | -0.001 (0.001) | | **-0.002** (0.001) | | **-0.001** (0.001) | |  |
| Control*Temp | **-0.002** (0.001) | **-0.003** (0.001) | | **-0.003** (0.001) | | -0.001 (0.001) | |  |
| Random Effects |  |  | |  | |  | |  |
| Intercept | 0.057 (15%) | 0.131 (49%) | | 0.127 (18.2%) | | 0.167 (17.3%) | |  |
| Residual | 1.116 (8.4%) | 1.878 (5%) | | 2.226 (4.2%) | | 1.885 (3.3%) | |  |
|  |  |  | |  | |  | |  |
|  |  |  | |  | |  | |  |
|  | Subjective  Well-Being | Physical Health | | Mental Health | | Life Satisfaction | |  |
|  |  |  | |  | |  | |  |
| Fixed Effects | **4.628** (0.060) | **4.838** (0.115) | | **4.845** (0.091) | | **4.536** (0.103) | |  |
| Intercept | **4.575** (0.050) | **4.778** (0.075) | | **4.753** (0.075) | | **4.457** (0.084) | |  |
| Age | **0.016** (0.001) | **-0.017** (0.001) | | 0.011 (0.001) | | **0.007** (0.001) | |  |
| Temporal Distance | **0.010** (0.002) | **0.011** (0.002) | | **0.013** (0.002) | | **0.006** (0.002) | |  |
| Valence | **0.170** (0.010) | 0.143 (0.013) | | 0.235 (0.142) | | **0.207** (0.013) | |  |
| Valence*Age | -0.001 (0.000) | -0.000 (0.000) | | **-0.002** (0.001) | | **-0.001** (0.000) | |  |
| Valence*Temp | **-0.005** (0.001) | -0.006 (0.001) | | **-0.006** (0.001) | | **-0.004** (0.001) | |  |
| Random Effects |  |  | |  | |  | |  |
| Intercept | 0.044 (34%) | 0.103 (60%) | | 0.102 (34.6%) | | 0.132 (34.7%) | |  |
| Residual | 1.097 (9.9%) | 1.871 (5.4%) | | 2.186 (5.9%) | | 1.866 (4.4%) | |  |
|  |  |  | |  | |  | |  |
|  |  |  | |  | |  | |  |
| Fixed effects | **4.628** (0.060) | **4.838** (0.115) | | **4.845** (0.091) | | **4.536** (0.103) | |  |
| Intercept | **4.611** (0.048) | **4.806** (0.083) | | **4.815** (0.078) | | **4.518** (0.090) | |  |
| Age | **0.014** (0.001) | **-0.018** (0.001) | | 0.008 (0.001) | | **0.005** (0.001) | |  |
| Temporal Distance | **0.010** (0.002) | **0.011** (0.002) | | **0.013** (0.002) | | **0.006** (0.002) | |  |
| Strain | **-0.152** (0.008) | **-0.106** (0.010) | | **-0.187** (0.011) | | **-0.129** (0.011) | |  |
| Strain*Age | 0.000 (0.000) | -0.001 (0.001) | | -0.000 (0.001) | | 0.001 (0.001) | |  |
| Strain*Temp | **0.005** (0.001) | **0.006** (0.001) | | **0.008** (0.001) | | **0.004** (0.001) | |  |
| Random Effects |  |  | |  | |  | |  |
| Intercept | 0.042 (37%) | 0.128 (50%) | | 0.113 (27.7%) | | 0.154 (23.8%) | |  |
| Residual | 1.088 (10.7%) | 1.876 (5.1%) | | 2.186 (5.9%) | | 1.894 (2.9%) | |  |

*Note.* Fixed effects: Standard errors in parentheses. Significant effects in bold (*p* <.05). Random effects: ∆R^2^ in parentheses.

| Table S8 |  | |  |  | |  |  |
| --- | --- | --- | --- | --- | --- | --- | --- |
| ANOVA Comparison of Models with and without Random Slopes | | | | | | | |
|  | AIC | BIC | | | Log-Likelihood | p | |
| Subjective Well-Being |  |  | | |  |  | |
| Anticipation | 20029.55 | 20084.0 | | | -10006.774 |  | |
| Random Slope |  |  | | |  |  | |
| Anticipation*Age | 20019.88 | 20135.6 | | | -9992.941 | .001 | |
| Anticipation*Temp | --- | --- | | | --- | --- | |
|  |  |  | | |  |  | |
| Normativity | 20016.67 | 20071.13 | | | -10000.334 |  | |
| Random Slope |  |  | | |  |  | |
| Normativity*Age | 20016.33 | 20132.06 | | | -9991.167 | .03 | |
| Normativity*Temp | --- | --- | | | --- | --- | |
|  |  |  | | |  |  | |
| Control | 19838.60 | 19893.06 | | | -9911.300 |  | |
| Random Slope |  |  | | |  |  | |
| Control*Age | 19820.31 | 19936.03 | | | -9893.157 | <.001 | |
| Control*Temp | --- | --- | | | --- | --- | |
|  |  |  | | |  |  | |
| Valence | 19726.16 | 19780.62 | | | -9855.081 |  | |
| Random Slope |  |  | | |  |  | |
| Valence*Age | 19703.45 | 19819.17 | | | -9834.724 | <.001 | |
| Valence*Temp | 19697.58 | 19813.30 | | | -9831.790 | <.001 | |
|  |  |  | | |  |  | |
| Strain | 19665.34 | 19719.79 | | | -9824.669 |  | |
| Random Slope |  |  | | |  |  | |
| Strain*Age | 19663.20 | 19778.93 | | | -9814.602 | .02 | |
| Strain*Temp | 19652.11 | 19767.83 | | | -9809.053 | <.001 | |
| Physical Health |  |  | | |  |  | |
| Anticipation | 23434.94 | 23489.40 | | | -11709.47 |  | |
| Random Slope |  |  | | |  |  | |
| Anticipation*Age | 23418.89 | 23534.61 | | | -11692.44 | <.001 | |
| Anticipation*Temp | --- | --- | | | --- | --- | |
|  |  |  | | |  |  | |
| Normativity | 23429.30 | 23483.76 | | | -11706.65 |  | |
| Random Slope |  |  | | |  |  | |
| Normativity*Age | 23423.81 | 23539.53 | | | -11694.90 | .005 | |
| Normativity*Temp | --- | --- | | | --- | --- | |
|  |  |  | | |  |  | |
| Control | 23321.08 | 23375.53 | | | -11652.54 |  | |
| Random Slope |  |  | | |  |  | |
| Control*Age | 23270.85 | 23386.58 | | | -11618.43 | <.001 | |
| Control*Temp | 23210.04 | 23325.76 | | | -11588.02 | <.001 | |
|  |  |  | | |  |  | |
| Valence | 23294.19 | 23348.65 | | | -11639.10 |  | |
| Random Slope |  |  | | |  |  | |
|  | AIC | BIC | | | Log-Likelihood | p | |
| Valence*Age | 23259.67 | 23375.40 | | | -11612.84 | <.001 | |
| Valence*Temp | 23215.63 | 23331.36 | | | -11590.82 | <.001 | |
|  |  |  | | |  |  | |
| Strain | 23313.00 | 23367.46 | | | -11648.50 |  | |
| Random Slope |  |  | | |  |  | |
| Strain*Age | 23290.65 | 23406.38 | | | -11628.33 | <.001 | |
| Strain*Temp | --- | --- | | | --- | --- | |
|  |  |  | | |  |  | |
| Mental Health |  |  | | |  |  | |
| Anticipation | 24601.37 | 24655.83 | | | -12292.69 |  | |
| Random Slope |  |  | | |  |  | |
| Anticipation*Age | 24575.81 | 24691.53 | | | -12270.90 | <.001 | |
| Anticipation*Temp | --- | --- | | | --- | --- | |
|  |  |  | | |  |  | |
| Normativity | 24604.56 | 24659.02 | | | -12294.28 |  | |
| Random Slope |  |  | | |  |  | |
| Normativity*Age | 24597.81 | 24713.53 | | | -12281.91 | .003 | |
| Normativity*Temp | --- | --- | | | --- | --- | |
|  |  |  | | |  |  | |
| Control | 24455.23 | 24509.69 | | | -12219.61 |  | |
| Random Slope |  |  | | |  |  | |
| Control*Age | 24440.36 | 24556.08 | | | -12203.18 | <.001 | |
| Control*Temp | 24312.28 | 24428.00 | | | -12139.14 | <.001 | |
|  |  |  | | |  |  | |
| Valence | 24329.77 | 24384.23 | | | -12156.89 |  | |
| Random Slope |  |  | | |  |  | |
| Valence*Age | 24310.59 | 24426.32 | | | -12138.30 | <.001 | |
| Valence*Temp | 24312.28 | 24428.00 | | | -12139.14 | <.001 | |
|  |  |  | | |  |  | |
| Strain | 24332.13 | 24386.58 | | | -12158.06 |  | |
| Random Slope |  |  | | |  |  | |
| Strain*Age | 24311.14 | 24426.86 | | | -12138.57 | <.001 | |
| Strain*Temp | 24413.32 | 24529.04 | | | -12189.66 | <.001 | |
|  |  |  | | |  |  | |
| Life Satisfaction |  |  | | |  |  | |
| Anticipation | 23517.11 | 23571.57 | | | -11750.56 |  | |
| Random Slope |  |  | | |  |  | |
| Anticipation*Age | 23482.62 | 23598.34 | | | -11724.31 | <.001 | |
| Anticipation*Temp | 23498.07 | 23613.79 | | | -11732.03 | <.001 | |
|  |  |  | | |  |  | |
| Normativity | 23510.26 | 23564.72 | | | -11747.13 |  | |
| Random Slope |  |  | | |  |  | |
| Normativity*Age | 23499.45 | 23615.17 | | | -11732.73 | <.001 | |
| Normativity*Temp | --- | --- | | | --- | --- | |
|  |  |  | | |  |  | |
| Control | 23350.72 | 23405.18 | | | -11667.36 |  | |
| Random Slope |  |  | | |  |  | |
| Control*Age | 23316.30 | 23432.02 | | | -11641.15 | <.001 | |
|  | AIC | BIC | | | Log-Likelihood | p | |
| Control*Temp | 23315.16 | 23430.89 | | | -11640.58 | <.001 | |
|  |  |  | | |  |  | |
| Valence | 23280.15 | 23334.61 | | | -11632.08 |  | |
| Random Slope |  |  | | |  |  | |
| Valence*Age | 23258.19 | 23373.92 | | | -11612.10 | <.001 | |
| Valence*Temp | 23266.27 | 23381.99 | | | -11616.14 | <.001 | |
|  |  |  | | |  |  | |
| Strain | 23380.18 | 23434.63 | | | -11682.09 |  | |
| Random Slope |  |  | | |  |  | |
| Strain*Age | 23359.83 | 23475.55 | | | -11662.92 | <.001 | |
| Strain*Temp | 23371.29 | 23487.01 | | | -11668.65 | .002 | |

*Note.* Degrees of freedom = 8, 17. AIC = Akakike information criterion; BIC = Bayesian information criterion; -- = no fit.
